# Supplementary material for: Time‑resolved multi-omic analysis of paclitaxel exposure in human iPSC‑derived sensory neurons unveils mechanisms of chemotherapy‑induced peripheral neuropathy
Source: Cell Death Dis. 2026 Feb 10;17(1):211. doi: 10.1038/s41419-026-08445-2 (PMC12921266; doi:10.1038/s41419-026-08445-2)
Supplement: Supplementary file 15 — Dataset 1. [file 41419_2026_8445_MOESM15_ESM.zip › CDD_Open_Data/04_RNA_Seq/Timeline_panel_plots_etc_2023-01-23_1609.zip!/Panel_Plots_etc/Evidence_Plots/72h/evidence_plot_standard contrasts_msigdb_hallmark_72h_PTX_vs_DMSO_ID0_M5944_HALLMARK_ANGIOGENESIS.pdf]

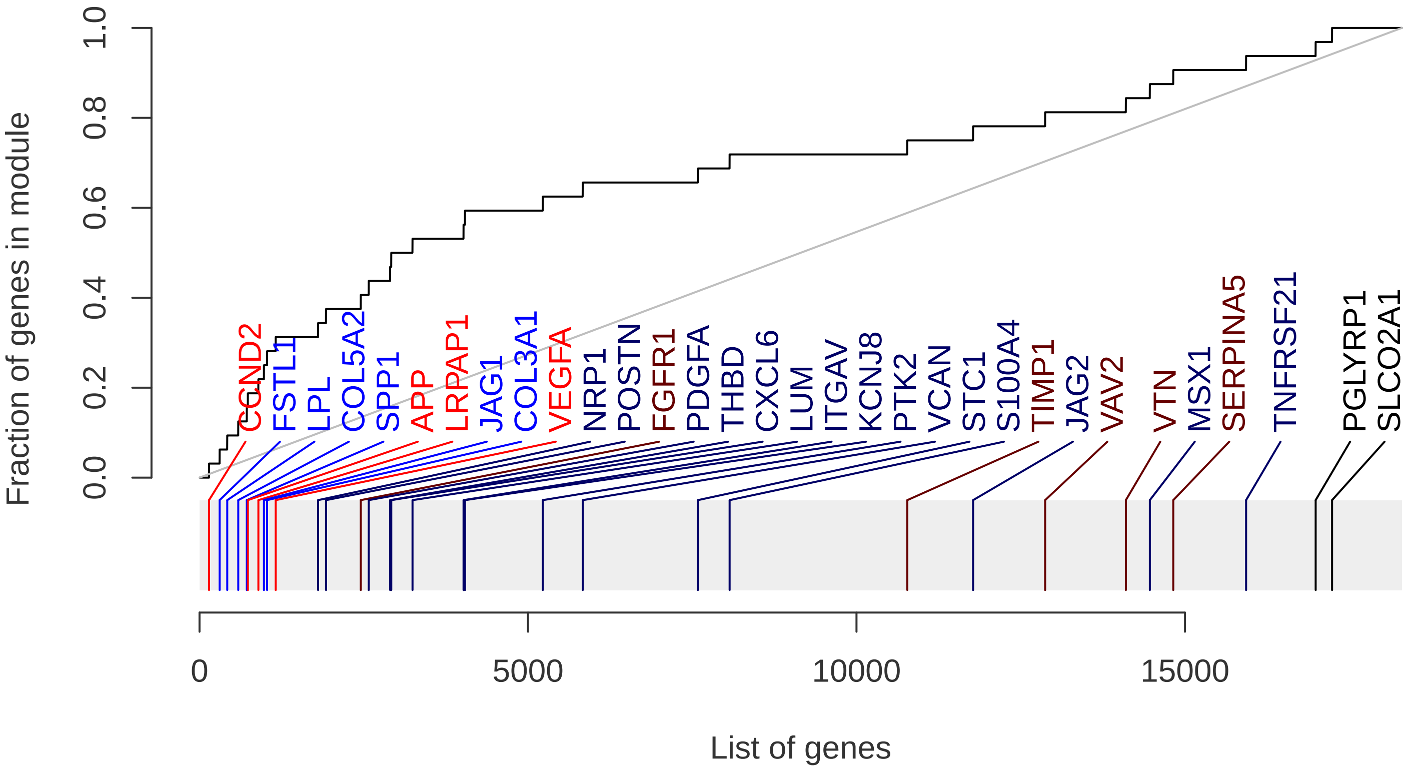

Fraction of genes in module
0.0
0.2
0.4
0.6
0.8
1.0
CCND2
FSTL1
LPL
COL5A2
SPP1
APP
LRPAP1
JAG1
COL3A1
VEGFA
NRP1
POSTN
FGFR1
PDGFA
THBD
CXCL6
LUM
ITGAV
KCNJ8
PTK2
VCAN
STC1
S100A4
TIMP1
JAG2
VAV2
VTN
MSX1
SERPINA5
TNFRSF21
PGLYRP1
SLCO2A1
0
5000
10000
15000
List of genes
